# Supplementary material for: Modeling glioblastoma heterogeneity as a dynamic network of cell states
Source: Mol Syst Biol. 2021 Sep 16;17(9):e10105. doi: 10.15252/msb.202010105 (PMC8444284; doi:10.15252/msb.202010105)
Supplement: Supplementary file 6 — Source Data for Figure 5 [file MSB-17-e10105-s004.zip › Figure5A_sourcedata/GSEA_3071/hallmarks_stateV.GseaPreranked.1623742546329/HALLMARK_INTERFERON_GAMMA_RESPONSE.html]

Details for gene set HALLMARK\_INTERFERON\_GAMMA\_RESPONSE[GSEA]

|  || Dataset | state43071 |
| Phenotype | NoPhenotypeAvailable |
| Upregulated in class | na\_neg |
| GeneSet | HALLMARK\_INTERFERON\_GAMMA\_RESPONSE |
| Enrichment Score (ES) | -0.49847102 |
| Normalized Enrichment Score (NES) | -2.5591962 |
| Nominal p-value | 0.0 |
| FDR q-value | 0.0 |
| FWER p-Value | 0.0 |
Table: GSEA Results Summary

  

Fig 1: Enrichment plot: HALLMARK\_INTERFERON\_GAMMA\_RESPONSE      
 Profile of the Running ES Score & Positions of GeneSet Members on the Rank Ordered List

  

| PROBE | GENE SYMBOL | GENE\_TITLE | RANK IN GENE LIST | RANK METRIC SCORE | RUNNING ES | CORE ENRICHMENT || 1 | CDKN1A |  |  | 363 | 0.993 | -0.0437 | No |
| 2 | SRI |  |  | 599 | 0.801 | -0.0680 | No |
| 3 | CASP3 |  |  | 1057 | 0.605 | -0.1397 | No |
| 4 | METTL7B |  |  | 1609 | 0.458 | -0.2332 | No |
| 5 | PDE4B |  |  | 1660 | 0.444 | -0.2313 | No |
| 6 | ISOC1 |  |  | 1695 | 0.437 | -0.2265 | No |
| 7 | C1R |  |  | 1696 | 0.437 | -0.2152 | No |
| 8 | USP18 |  |  | 1885 | 0.406 | -0.2406 | No |
| 9 | ST8SIA4 |  |  | 2207 | 0.361 | -0.2927 | No |
| 10 | CMKLR1 |  |  | 2245 | 0.357 | -0.2905 | No |
| 11 | IL7 |  |  | 2500 | 0.322 | -0.3307 | No |
| 12 | RAPGEF6 |  |  | 2580 | 0.313 | -0.3377 | No |
| 13 | AUTS2 |  |  | 2613 | 0.309 | -0.3359 | No |
| 14 | SPPL2A |  |  | 3154 | 0.255 | -0.4325 | No |
| 15 | PSME1 |  |  | 3202 | -0.251 | -0.4350 | No |
| 16 | ZNFX1 |  |  | 3297 | -0.266 | -0.4461 | No |
| 17 | DDX60 |  |  | 3304 | -0.266 | -0.4404 | No |
| 18 | TNFAIP2 |  |  | 3314 | -0.269 | -0.4351 | No |
| 19 | PARP12 |  |  | 3343 | -0.274 | -0.4334 | No |
| 20 | MX2 |  |  | 3344 | -0.274 | -0.4263 | No |
| 21 | PTPN2 |  |  | 3499 | -0.300 | -0.4480 | No |
| 22 | PNPT1 |  |  | 3513 | -0.302 | -0.4427 | No |
| 23 | IFITM2 |  |  | 3585 | -0.312 | -0.4482 | No |
| 24 | CASP1 |  |  | 3648 | -0.323 | -0.4517 | No |
| 25 | TNFSF10 |  |  | 3651 | -0.323 | -0.4437 | No |
| 26 | TNFAIP3 |  |  | 3691 | -0.330 | -0.4426 | No |
| 27 | TAPBP |  |  | 3712 | -0.333 | -0.4378 | No |
| 28 | RIPK2 |  |  | 3867 | -0.362 | -0.4579 | No |
| 29 | IFIT3 |  |  | 3874 | -0.362 | -0.4497 | No |
| 30 | HIF1A |  |  | 4130 | -0.417 | -0.4877 | Yes |
| 31 | RBCK1 |  |  | 4165 | -0.428 | -0.4831 | Yes |
| 32 | SOCS3 |  |  | 4191 | -0.435 | -0.4766 | Yes |
| 33 | MTHFD2 |  |  | 4212 | -0.444 | -0.4690 | Yes |
| 34 | NFKB1 |  |  | 4313 | -0.476 | -0.4758 | Yes |
| 35 | PSMB8 |  |  | 4318 | -0.477 | -0.4642 | Yes |
| 36 | B2M |  |  | 4341 | -0.485 | -0.4559 | Yes |
| 37 | IL15 |  |  | 4346 | -0.486 | -0.4441 | Yes |
| 38 | IFI44L |  |  | 4387 | -0.497 | -0.4389 | Yes |
| 39 | PSME2 |  |  | 4422 | -0.508 | -0.4323 | Yes |
| 40 | IFITM3 |  |  | 4424 | -0.509 | -0.4193 | Yes |
| 41 | PSMB9 |  |  | 4503 | -0.543 | -0.4202 | Yes |
| 42 | CASP4 |  |  | 4551 | -0.565 | -0.4145 | Yes |
| 43 | ISG15 |  |  | 4586 | -0.579 | -0.4061 | Yes |
| 44 | ARL4A |  |  | 4593 | -0.582 | -0.3922 | Yes |
| 45 | PSMB2 |  |  | 4594 | -0.583 | -0.3771 | Yes |
| 46 | TXNIP |  |  | 4618 | -0.604 | -0.3658 | Yes |
| 47 | PSMB10 |  |  | 4628 | -0.608 | -0.3519 | Yes |
| 48 | SAMD9L |  |  | 4638 | -0.613 | -0.3377 | Yes |
| 49 | PSMA3 |  |  | 4650 | -0.618 | -0.3238 | Yes |
| 50 | NCOA3 |  |  | 4720 | -0.661 | -0.3199 | Yes |
| 51 | PFKP |  |  | 4734 | -0.666 | -0.3052 | Yes |
| 52 | EPSTI1 |  |  | 4742 | -0.672 | -0.2891 | Yes |
| 53 | CD274 |  |  | 4743 | -0.673 | -0.2717 | Yes |
| 54 | MVP |  |  | 4760 | -0.686 | -0.2570 | Yes |
| 55 | TNFAIP6 |  |  | 4764 | -0.688 | -0.2398 | Yes |
| 56 | PML |  |  | 4765 | -0.689 | -0.2220 | Yes |
| 57 | PIM1 |  |  | 4783 | -0.699 | -0.2072 | Yes |
| 58 | APOL6 |  |  | 4798 | -0.713 | -0.1914 | Yes |
| 59 | MT2A |  |  | 4799 | -0.714 | -0.1729 | Yes |
| 60 | BPGM |  |  | 4904 | -0.818 | -0.1717 | Yes |
| 61 | NAMPT |  |  | 4906 | -0.820 | -0.1507 | Yes |
| 62 | NMI |  |  | 5098 | -1.142 | -0.1576 | Yes |
| 63 | IRF1 |  |  | 5100 | -1.155 | -0.1280 | Yes |
| 64 | SOCS1 |  |  | 5184 | -1.475 | -0.1057 | Yes |
| 65 | CFH |  |  | 5257 | -2.141 | -0.0641 | Yes |
| 66 | SSPN |  |  | 5284 | -2.752 | 0.0021 | Yes |
Table: GSEA details [plain text format]

  

Fig 2: HALLMARK\_INTERFERON\_GAMMA\_RESPONSE: Random ES distribution      
 Gene set null distribution of ES for **HALLMARK\_INTERFERON\_GAMMA\_RESPONSE**
